# Controlling the Spatiotemporal Release of Nerve Growth Factor by Chitosan/Polycaprolactone Conduits for Use in Peripheral Nerve Regeneration

**DOI:** 10.3390/ijms23052852

**Published:** 2022-03-05

**Authors:** Katarzyna Nawrotek, Monika Kubicka, Justyna Gatkowska, Marek Wieczorek, Sylwia Michlewska, Adrian Bekier, Radosław Wach, Karolina Rudnicka

**Affiliations:** 1 Department of Environmental Engineering, Faculty of Process and Environmental Engineering, Lodz University of Technology, 213 Wolczanska Street, 90-924 Lodz, Poland; monika.kubicka@p.lodz.pl; 2 Department of Molecular Microbiology, Faculty of Biology and Environmental Protection, University of Lodz, 12/16 Banacha Street, 90-237 Lodz, Poland; justyna.gatkowska@biol.uni.lodz.pl (J.G.); adrian.bekier@biol.uni.lodz.pl (A.B.); 3 Department of Neurobiology, Faculty of Biology and Environmental Protection, University of Lodz, 141/143 Pomorska Street, 90-236 Lodz, Poland; marek.wieczorek@biol.uni.lodz.pl; 4 Laboratory of Microscopic Imaging and Specialized Biological Techniques, Faculty of Biology and Environmental Protection, University of Lodz, 12/16 Banacha Street, 90-237 Lodz, Poland; sylwia.michlewska@biol.uni.lodz.pl; 5 Faculty of Chemistry, Institute of Applied Radiation Chemistry, Lodz University of Technology, 15 Wroblewskiego Street, 93-590 Lodz, Poland; radoslaw.wach@p.lodz.pl; 6 Department of Immunology and Infectious Biology, Faculty of Biology and Environmental Protection, University of Lodz, 12/16 Banacha Street, 90-237 Lodz, Poland

**Keywords:** electrodeposition, 3D printing, chitosan, conduit, peripheral nerve regeneration

## Abstract

Tubular polymeric structures have been recognized in the treatment of peripheral nerves as comparable to autologous grafting. The best therapeutic outcomes are obtained with conduits releasing therapeutic molecules. In this study, a new approach for the incorporation of biologically active agent-loaded microspheres into the structure of chitosan/polycaprolactone conduits was developed. The support of a polycaprolactone helix formed by 3D melt extrusion was coated with dopamine in order to adsorb nerve growth factor-loaded microspheres. The complex analysis of the influence of process factors on the coverage efficiency of polycaprolactone helix by nerve grow factor-loaded microspheres was analyzed. Thus, the PCL helix characterized with the highest adsorption of microspheres was subjected to nerve growth factor release studies, and finally incorporated into chitosan hydrogel deposit through the process of electrophoretic deposition. It was demonstrated by chemical and physical tests that the chitosan/polycaprolactone conduit meets the requirements imposed on peripheral nerve implants, particularly mimicking mechanical properties of surrounding soft tissue. Moreover, the conduit may support regrowing nerves for a prolonged period, as its structure and integrity persist upon incubation in lysozyme-contained PBS solution up to 28 days at body temperature. In vitro cytocompatibility toward mHippoE-18 embryonic hippocampal cells of the chitosan/polycaprolactone conduit was proven. Most importantly, the developed conduits stimulate axonal growth and support monocyte activation, the latter is advantageous especially at early stages of nerve regeneration. It was demonstrated that, through the described approach for controlling spatiotemporal release of nerve growth factors, these biocompatible structures adjusted to the specific peripheral nerve injury case can be manufactured.

## 1. Introduction

A peripheral nerve injury (PNI) relates to damage, crushing, or transection of the peripheral nerve. It is estimated that 3% of all critical trauma patients present some form of nerve defect [1,2]. Moreover, PNIs can result from degenerative changes or inflammatory diseases [3,4]. Peripheral nerves are key connections among the brain, spinal cord, and body. This highly complex network provides control over sensation, movement, and motor coordination. Treatment of damaged peripheral nerve tissue may be pharmacologic, by transplantation (i.e., autografting, allografting, or xenografting) or by the implantation of nerve guidance conduits (NGCs) to bridge the nerve stumps, providing room and environment for nerve regeneration [5,6,7,8]. Conventional pharmacologic therapy is performed in the early stages of damage caused by inflammation. Transplantation is a complex procedure, with a risk of graft rejection, as well as complications (e.g., infection). Therefore, innovative solutions allowing for re-generation of damaged tissues with the use of NGCs are being sought.

Nerve guidance conduits should serve as a platform stimulating nerve regeneration at lesion sites [9]. Their main functions are to induce the proper orientation of axon growth from the proximal end of the nerve, to accumulate growth factors secreted by damaged nerve stumps within its lumen, and to prevent scar tissue growth within the lesion site [10,11,12,13]. In addition to biocompatibility, a desired characteristic of the conduits is biodegradability [14,15]. The guidance of axons is evoked by controlled release of biologically active substances (e.g., neurotrophic factors) from the structure of the NGC [16,17]. Preferentially, the active agents should be incorporated in the NGC in a gradient manner [18,19]. Neurotrophic factors are characterized with a short in vivo half-life. Moreover, they impede axonal regeneration when used in supraphysiological doses. Therefore, different approaches to counteract these phenomena have been developed [20]. One of the most studied ones is encapsulation of hydrophilic molecules such as proteins or nucleic acids within a polymer [21,22,23]. Encapsulation requires proper selection of polymeric composition and optimization of synthesis parameters in order to ensure the highest possible encapsulation efficiency while maintaining the biological activity of the encapsulated biomolecule [24]. In the last few years, numerous publications have mentioned delivery systems of bone morphogenetic protein 2 (BMP2) or growth factors (GF) using polymeric particles [25]. Polymeric microspheres seem to be a promising method to control the release of biologically active substances.

A technology for obtaining chitosan/polycaprolactone nerve guidance conduits, which employs polymer extrusion and electrophoretic deposition, was developed in our laboratory [8]. The fabricated structures were made of two layers: polycaprolactone (PCL) helix and chitosan (CH) hydrogel deposit. In this work, the surface of the PCL helix was covered by dopamine. Such modification allowed the adsorption of microspheres; thus, it could serve as a platform for the controlled release of biologically active substances. Since the PCL helix can be characterized by different pitch, the concentration of active agents could be regulated. Moreover, the pitch can be constant or gradient. The first part of this work was to study the influence of different process parameters on the coverage efficiency of the PCL helix by nerve grow factor-loaded microspheres (NGF-µS), such as the influence of microsphere dimensions, the effect of dopamine and microsphere concentration, and the release profile of NGF from NGF-µS/PCL helices, determined by enzyme-linked immunosorbent assay (ELISA), was monitored over a period of 14 days. In the second part, the PCL helix covered by nerve grow factor-loaded microparticles was incorporated into the CH hydrogel by electrophoretic deposition. Subsequently, chemical, physical, and mechanical properties of conduits and their stability in enzymatic environment were evaluated. Lastly, in vitro cell studies were conducted for cytocompatibility evaluation and to assess axonal growth of hippocampal cells and nuclear factor (NF-κB) activation in THP1--Blue™ NF-κB monocytes. It is anticipated that the obtained structures will find application as implants in tissue engineering of the peripheral nervous system. The developed method of incorporation of polymeric microspheres into the structure of chitosan/polycaprolactone nerve guidance conduits may contribute to progress in regeneration of damaged nervous tissue by facilitating axonal guidance inside the conduit lumen.

## 2. Results and Discussion

### 2.1. Characterization of Microspheres and Their Adsorption to Polycaprolactone Filament

The first step of the work aimed at covering the PCL helix with dopamine in order to allow adsorption of NGF-loaded microspheres (NGF-µS). Poly(lactide-*co*-glycolide) (PLGA) encapsulating NGF microcapsules were prepared by the water-in-oil-in-water (W/O/W) method with poly(vinyl alcohol) (PVA) as a stabilizer in the external aqueous phase as previously described [26,27]. The main advantage of the W/O/W dispersion method is the maintenance of bioactivity of the encapsulated biologically active substances. The properties of microspheres produced by this method depend on the concentrations of PLGA and stabilizing polymer, emulsification volumes, emulsification energy applied, viscosities of the different phases, and additives [28].

**Figure 1:**
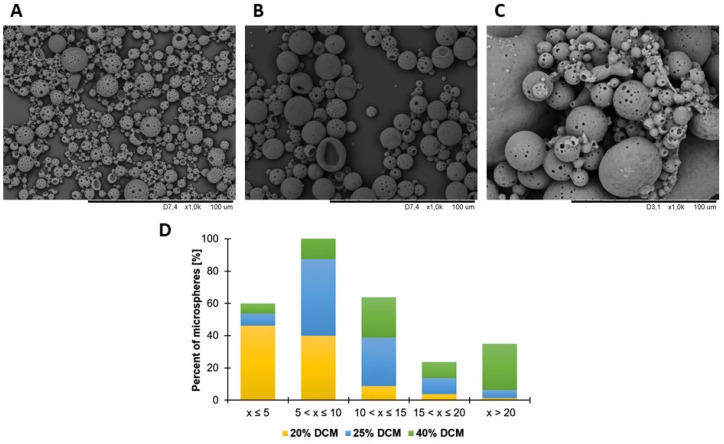
Scanning electron microscope images of microspheres obtained by water-in-oil-in-water method using (**A**) 20%, (**B**) 25%, and (**C**) 40% poly(lactide-*co*-glycolide) in dichloromethane. (**D**) Variety of microsphere diameters depending on concentration of PLGA in DCM.

For optimization studies, microspheres were prepared without NGF (µS). Firstly, the influence of microsphere diameter on adsorption efficiency was determined. For this purpose, three concentrations of poly(lactide-*co*-glycolide) in dichloromethane (20%, 25%, and 40%) were applied. Figure 1 shows the variety of microsphere diameters depending on concentration of PLGA in dichloromethane (DCM).

**Figure 2:**
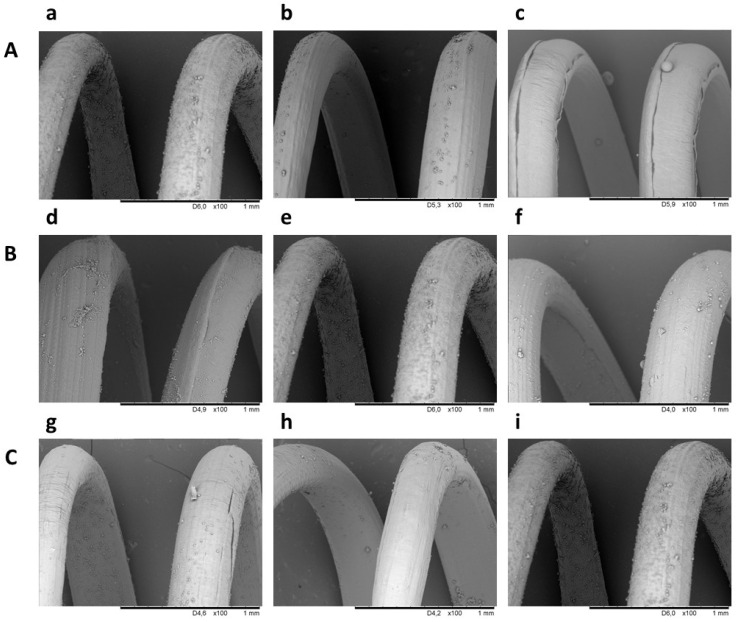
Scanning electron microscope images illustrating the influence of (**A**) concentration of PLGA in DCM ((a) 20%, (b) 25%, and (c) 40%), (**B**) dopamine concentration ((d) 0.2%, (e) 0.4%, and (f) 0.6%), and (**C**) microsphere concentration ((g) 1.19%, (h) 1.50%, and (i) 2.34%) on microsphere adsorption efficiency to PCL helix.

The PCL helices covered by dopamine showed different adsorption efficiency of microspheres depending on their average diameters (Figure 2A). The highest adsorption was observed for microspheres prepared from solution containing 20% PLGA in DCM. In this case, 46.3% of microspheres prepared applying these conditions were characterized with a diameter not higher than 5 µm. This concentration was used in the next optimization steps. In the next step of the work, the influence of dopamine concentration on microsphere adsorption was studied. Figure 2B shows adsorption of microspheres on PCL helices modified in a solution of 0.2%, 0.4%, and 0.6% dopamine hydrochloride. The highest adsorption was observed for PCL filament modified in a solution of 0.4% dopamine hydrochloride, and this condition was used in the next step. The final optimization step aimed at determining the influence of microsphere concentration on adsorption efficiency. A higher microsphere concentration resulted in a higher adsorption (Figure 2C).

On the basis of the optimization studies, the conditions leading to the highest adsorption efficiency were determined. Firstly, the microspheres should be prepared from a solution containing 20% PLGA in DCM. Secondly, the surface of the PCL helix should be modified in solution containing 0.4% dopamine hydrochloride. Lastly, the modified PCL helix should be exposed to the solution containing 2.3% microspheres.

### 2.2. Kinetics of NGF Release from NGF-µS/PCL Helix

**Figure 3:**
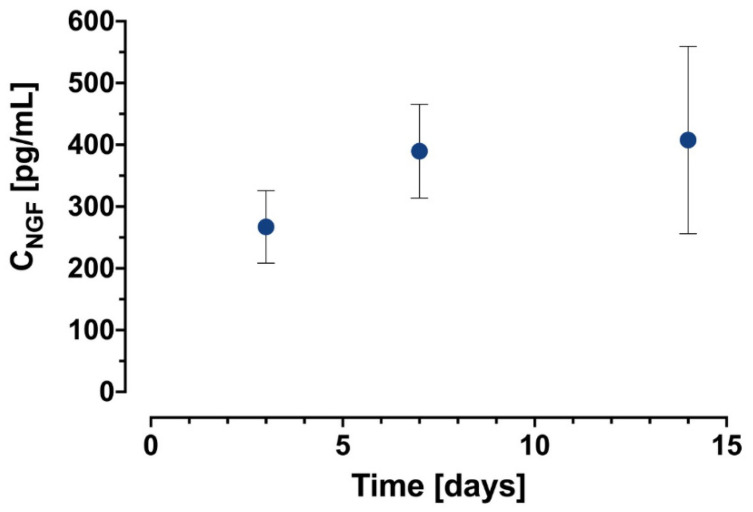
Cumulative release profile of NGF (pg/cm of NGF-µS/PCL helix) over a 14 day period from µS/PCL helix.

The NGF-µS/PCL helix was prepared under the conditions selected in the previous experiment. The release profile of NGF from NGF-µS/PCL helices, determined by NGF ELISA, over a period of 14 days is shown in Figure 3. After an initial burst, 267.0 ± 58.6 pg/cm of NGF from 1 cm of NGF-µS/PCL helix was released. After 14 days of the study, 407.8 ± 151.7 pg of encapsulated NGF from 1 cm of NGF-µS/PCL helix was released. The kinetics of the NGF release corresponds to that obtained in the literature [19].

The release of therapeutic molecules from PLGA microspheres is dependent on the physicochemical characteristics of the encapsulated molecule and created interaction (i.e., ionic, hydrophobic, adsorption, etc.) with the PLGA matrix during preparation [28]. Therapeutic molecule release kinetics is also dependent on the molecular weight of the PLGA used. The lower molecular weight of PLGA results in faster release [29]. It is a consequence of less physical entanglement between the polymer chains and more rapid pore formation during initial hydration and quick hydrolytic degradation of 50:50 lactide–glycolide copolymer.

Therapeutic molecule release from PLGA microspheres prepared by the W/O/W method with PVA as a stabilizer in the external aqueous phase is governed by diffusion- and degradation-mediated release mechanisms [19]. The initial burst release is observed due to desorption of loosely attached therapeutic molecules in surface pores or near the surface of the microspheres. Upon losing the pore-filling or surface-bound proteins, the microsphere wall is characterized by an interconnecting porous network through which further therapeutic molecule release can take place.

### 2.3. Incorporation of Polycaprolactone Filament into Hydrogel Chitosan Deposit

**Figure 4:**
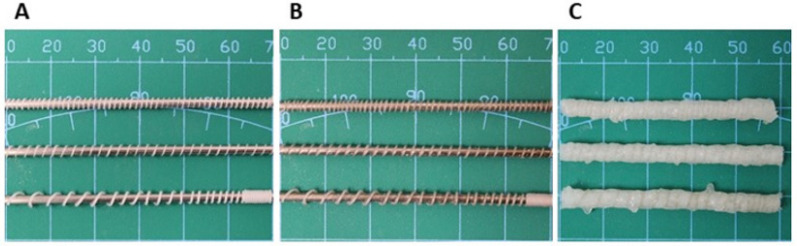
(**A**) PCL helix can be extruded with predefined geometry, constant or gradient pitch. (**B**) NGF-loaded microspheres can be successfully adsorbed to PCL helix covered by dopamine. (**C**) NGF-µS/PCL helix can be covered by chitosan hydrogel deposit in the process of electrophoretic deposition.

The method developed in our laboratory allows extruding the PCL fiber in helix form with predefined geometry (i.e., pitch, fiber diameter) (Figure 4A) [8]. Moreover, the pitch can be constant or gradient (Figure 4A). In the previous section, a study on the microsphere adsorption efficiency to PCL helix was presented. NGF-loaded microspheres can be efficiently adsorbed onto PCL helix covered by dopamine (Figure 4B). Furthermore, the NGF-µS/PCL helix can be covered by chitosan hydrogel deposit in the process of electrophoretic deposition to obtain a tubular conduit (Figure 4C).

### 2.4. Structural Characterization of the Chitosan/Polycaprolactone Implants

The electrodeposition process is carried out in a cylindrical reactor made of stainless steel. The NGF-µS/PCL helix is mounted onto a rod with predefined diameter corresponding to helix diameter, which serves as a cathode. The solution of electrically charged polymers is obtained by dissolving chitosan, hydroxyapatite, and sodium hyaluronate in an aqueous solution of lactic acid. Under the influence of the acidic environment, the amino groups of chitosan are protonated by hydronium ions (H_3_O^+^). Application of an electric current to the system results in the reduction of water at the surface of the cathode according to the following equation:

(1) $$2H_{2}O+2\bar{e}\to 2{\mathrm{OH}}^{-}+H_{2}.$$

The above reaction starts the alkalization of the area surrounding the cathode by increasing the amount of hydroxyl ions. At the same time, hydrogen is released. This phenomenon leads to deprotonation of chitosan amino groups according to the following formula:

(2) $$\mathrm{Chit}-{\mathrm{NH}}_{3}^{+}+{\mathrm{OH}}^{-}\to \mathrm{Chit}-{\mathrm{NH}}_{2}+H_{2}.$$

**Figure 5:**
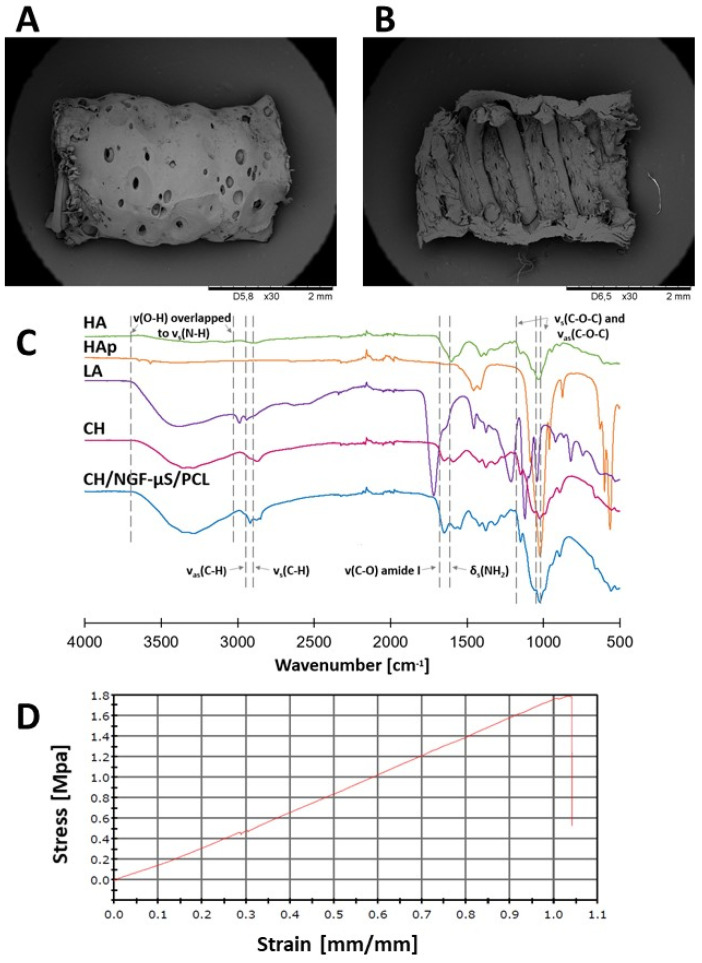
SEM images at 30× magnification of (**A**) longitudinal view and (**B**) inner sidewall view of CH/NGF-µS/PCL conduit. (**C**) FTIR spectrum of outer side of external surface of CH/NGF-µS/PCL conduit. (**D**) Example of stress–strain profile.

The structure of the formed chitosan hydrogel deposit was irregular and porous due to gas evolution during electrodeposition (Figure 5A). The ideal material for implants intended for peripheral nerve tissue engineering should be in the form of a membrane allowing the diffusion of oxygen and nutrients into the regeneration site from interstitial fluid. On the other hand, conduit walls should constitute a barrier to the infiltration of inflammatory cells into the conduit lumen. The obtained composition of chitosan hydrogel deposit is favorable for the release of NGF from the NGF-µS/PCL helix immersed in it.

In order to assess the interaction of compounds forming the chitosan hydrogel deposit, Fourier transform infrared spectroscopy (FTIR) spectroscopy was applied (Figure 5C). Chitosan moieties revealed peaks present at 1647 cm^−1^ (–C=O stretching mode), at 1580 cm^−1^ (–NH_2_ bending mode), and three in the range from 1020 to 1140 cm^−1^ (C–O–C stretching asymmetric and symmetric mode) [29]. The peaks characteristic for moieties of hydroxyapatite (PO_4_^3−^) could be distinguished at 1020, 961, 599, and 561 cm^−1^ [30]. The stretching mode of the –OH bond was denoted by a peak at 3562 cm^−1^. The FTIR spectrum of conduit external surface showed peaks characteristic for chitosan, hydroxyapatite, lactic acid, and hyaluronic acid [8]. Deformation of peaks at 1647 and 1580 cm^−1^ suggested the formation of new interactions between chitosan chains and hydroxyapatite-derived moieties. Moreover, the conduit surface showed weak signals at 1735 cm^−1^, which might indicate the presence of lactate ions. Characteristic bands for hydroxyapatite moieties could be observed at 960 cm^−1^ due to the presence of symmetric stretching vibrations of ν_1_(PO_4_^3−^).

Mean ultimate stress, mean ultimate strain, and Young’s modulus were calculated from the initial length and thickness of implants. An example of the stress–strain curve is presented in Figure 5D. The ultimate stress is measured as force per sample initial cross-sectional area at tensile failure, whereas the ultimate strain corresponds to the sample elongation achieved at the point of tensile failure divided by its initial length. Young’s modulus refers to the ability of a sample to withstand changes in length subjected to lengthwise tension. The ultimate stress, ultimate strain, and Young’s modulus of the CH/NGF-µS/PCL conduit were equal to 1.57 ± 0.50 MPa, 0.68 ± 0.22 mm/mm, and 1.85 ± 0.21 MPa, respectively.

The literature states that the mechanical properties of implants intended for peripheral nerve tissue should be referred to those of native peripheral nerve tissue. For example, the ultimate stress, ultimate strain, and Young’s modulus of acellular rat sciatic nerves are equal to 1.40 ± 0.29 MPa, 0.48 ± 0.12 mm/mm, and 0.580 ± 0.16 MPa, respectively [31]. On the basis of the obtained measurements, it can be concluded that the mechanical properties of the CH/NGF-µS/PCL conduit are sufficient to fulfil the requirements of peripheral nerve tissue engineering.

### 2.5. Kinetics of In Vitro Biodegradation

**Figure 6:**
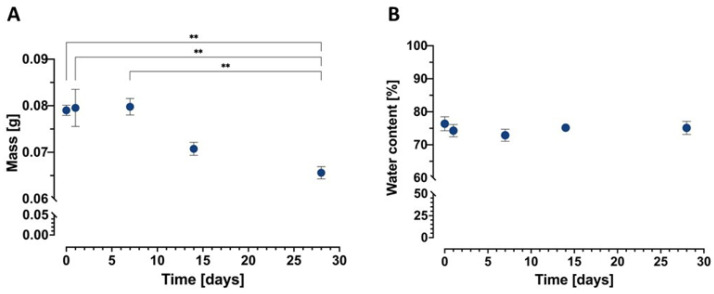
Change in (**A**) mass and (**B**) water content during incubation of CH/NGF-µS/PCL conduits in phosphate-buffered solution (pH 7.4) with lysozyme at 37 °C up to 28 days. Values with statistically significant differences are labeled by asterisks: ** *p* < 0.005.

The obtained conduits undergo mass loss after 7 days up to 28 days of incubation in phosphate-buffered solution (pH 7.4) with lysozyme at 37 °C (Figure 6A). One-way ANOVA indicated significant differences in the case of mass change, F_(4,20)_ = 8.664, *p* = 0.0003. Subsequent post hoc Tukey’s test indicated that the sample at day 28 differed from samples at days 0 (*p* < 0.003), 1 (*p* < 0.002), and 7 (*p* < 0.002). The kinetics of conduit mass loss upon storage in phosphate-buffered solution (PBS, pH 7.4) containing 1.5 mg/mL of lysozyme corresponds to that obtained in the literature [32]. Moreover, one-way ANOVA did not indicate significant changes in water content up to 28 days of conduit incubation, F_(4,20)_ = 2.469, *p* = 0.0779 (Figure 6B).

### 2.6. Cell Activity and Morphology

**Figure 7:**
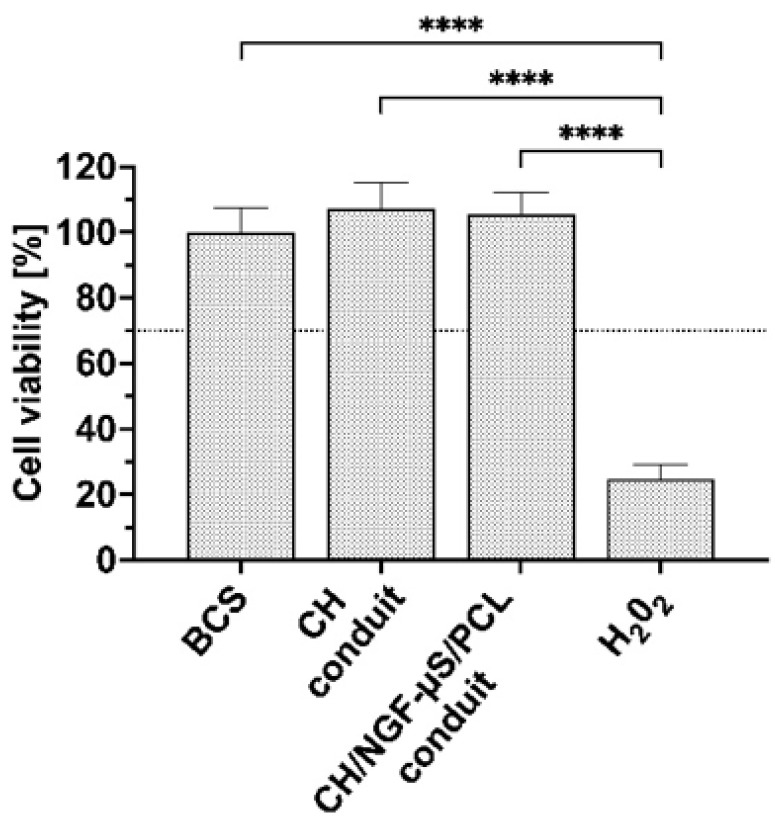
The viability of mHippoE-18 cells exposed for 24 h to CH and CH/NGF-µS/PCL conduits. Data are shown as the mean viability ± standard deviation (*n* = 6) compared to cells grown in the presence of BCS reference biomaterial (*n* = 6). Values with statistically significant differences are labeled by brackets and asterisks: **** *p* < 0.0001. Data were compared using an ordinary one-way ANOVA followed by Tukey’s multiple comparisons.

CH/NGF-µS/PCL conduits were tested for their cytocompatibility toward the mHippoE-18 cell line. Possible cytotoxicity was evaluated upon direct contact of the conduit with the cell monolayer after 24 h of incubation and expressed as the mean viability of cells ± SD in relation to cells cultured in the presence of a commercially available reference biomaterial Safety-Lok™ blood collection set (BCS). The results were compared with those obtained for a conduit made of chitosan hydrogel deposit (CH conduit). As shown in Figure 7, both tested conduits, similarly to the BCS reference material, had no cytotoxic activity toward mHippoE-18 cells, and the viability exceeded 100% for CH and CH/NGF-µS/PCL conduits: 107.3% ± 7.8% and 105.6% ± 6.6%, respectively. These findings are in line with the cytocompatibility of chitosan/PCL-based biomaterials obtained by other researchers [33,34]. In the case of the obtained CH/NGF-µS/PCL conduit, it was shown that this biomaterial candidate remains completely harmless (beyond 100% viability compared to control biomaterial) to more sensitive mHippoE-18 hippocampal cells. Moreover, one-way ANOVA indicated significant differences in the viability of mHippoE-18 cells, F_(3,20)_ = 212.2, *p* < 0.0001, and subsequent post hoc Tukey’s test indicated that only cells treated with H_2_O_2_ differed from samples BCS (*p* < 0.0001), CH conduit (*p* < 0.0001), and CH/NGF-µS/PCL conduits (*p* < 0.0001).

**Figure 8:**
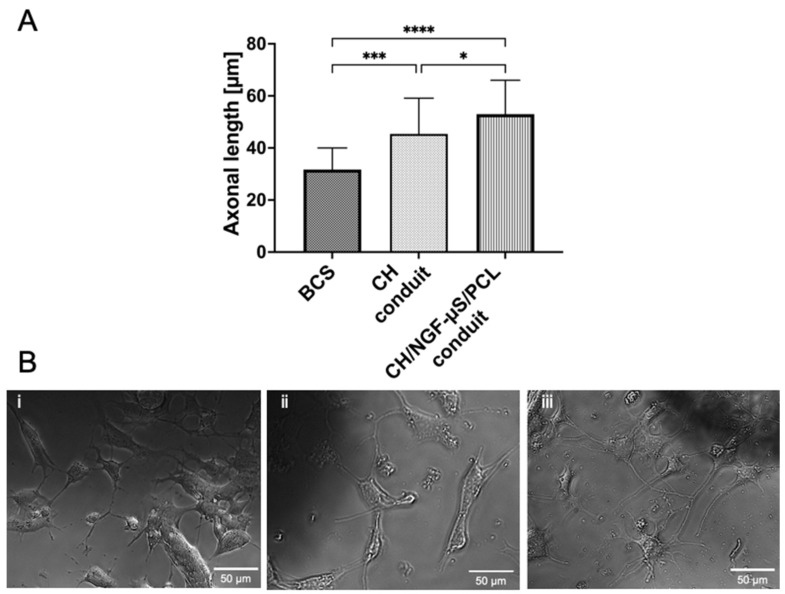
The influence of CH and CH/NGF-µS/PCL conduits on mHippoE-18 cells regarding their ability to stimulate axon elongation (**A**) and morphology (**B**) evaluated by light microscopy: mHippoE-18 cells grown in the presence of commercially available biomaterial BCS (i), CH conduit (ii), and CH/NGF-µS/PCL conduit (iii). Conduits are visible as darker areas in the pictures. Values with statistically significant differences are labeled by brackets and asterisks as follows: * *p* < 0.05, *** *p* < 0.001, **** *p* < 0.0001. Data were compared using an ordinary one-way ANOVA followed by Tukey’s multiple comparisons.

The influence of CH/NGF-µS/PCL conduit on mHippoE-18 cell morphology and axon elongation was also tested. The cells maintained their regular morphology growing in the presence of both CH and CH/NGF-µS/PCL conduits, as shown in the light microscope images (Figure 8(Bii, iii)), compared to cells propagated in the presence of commercially available control biomaterial—BCS (Figure 8(Bi)). Furthermore, a significant axon elongation was observed in the presence of CH/NGF-µS/PCL (56.00 ± 13.0 µm) and CH (45.4 ± 13.8 µm) conduits, when compared to BCS reference polymer (31.7 ± 8.3 µm) (Figure 8A). Furthermore, the CH/NGF-µS/PCL conduit was shown to induce the strongest axonal elongation; hippocampal cells in response to NGF-modified CH/PCL biomaterial produced significantly longer axons when compared to CH and BSC biomaterials. Moreover, it was shown that CH/NGF-µS/PCL conduit is not only cytocompatible, but also supports the increase in axonal length due to the presence of NGF.

The obtained results give strong evidence that the process of conduit production did not affect the biological activity of this neurotrophic polypeptide manifested by axonal elongation observed in the presented study and in the literature [35,36,37].

**Figure 9:**
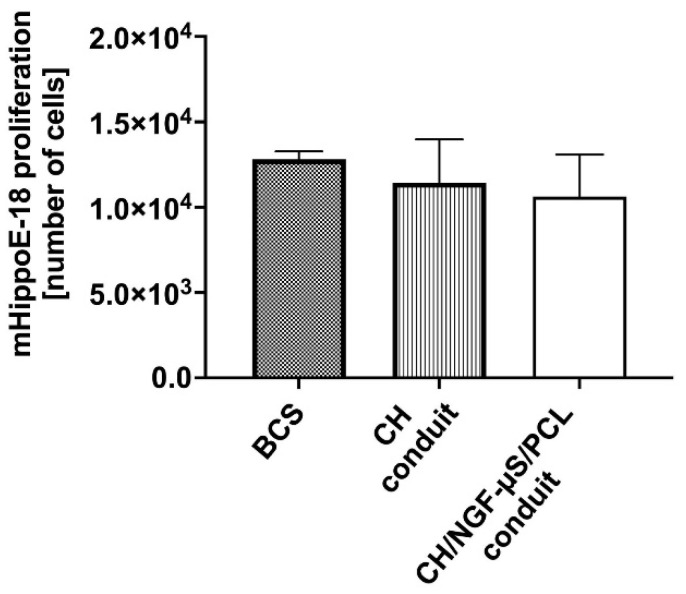
Proliferation of mHippoE-18 cells in response to CH and CH/NGF-µS/PCL conduits in comparison to proliferation rate in the presence of the reference material (BCS). Data are shown as a mean from three repeats with error bars that indicate standard deviation. Data were compared using an ordinary one-way ANOVA followed by Tukey’s multiple comparisons.

To address the question whether the obtained scaffolds support cell proliferation, the total DNA content in cells exposed to both CH and CH/NGF-µS/PCL conduits or maintained in the presence of commercially available biomaterial (BCS) was measured. One-way ANOVA did not indicate significant changes in the proliferation rate of mHippoE-18 in the presence of tested conduits and control biomaterial F_(2,6)_ = 0.8760, *p* = 0.4637 (Figure 9). The lack of proliferation might be explained by the observed changes in morphology, which was targeted on adhesion and axonal elongation, rather than cell expansion.

### 2.7. Conduit-Mediated NF-κB Activation

**Figure 10:**
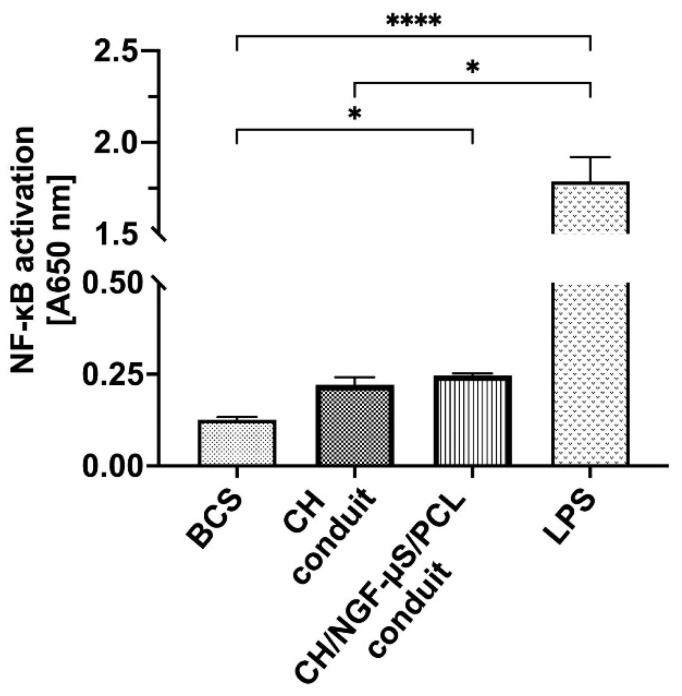
The NF-κB induction in THP1-Blue™ human monocytes exposed to CH or CH/NGF-µS/PCL conduits, BCS reference biomaterial (negative control), or *E. coli* lipopolysaccharide (LPS) at 0.25 IU (positive control). Data are shown as a mean from four repeats with error bars that indicate standard deviation. Values with statistically significant differences are labeled by brackets and asterisks as follows: * *p* < 0.05, **** *p* < 0.0001. Data were compared using a regular one-way ANOVA followed by Tukey’s multiple comparisons.

Using the THP1-Blue™ NF-κB reporter cell line, it was shown that CH/NGF-µS/PCL conduit and the commercial reference biomaterial intended for contact with body fluids did not induce NF-κB activation higher than that induced by a critical level of lipopolysaccharide (LPS, 0.25 IU/mL) allowed to remain in such biomaterials (Figure 10).

The LPS-induced activation was significantly higher when compared with BCS-treated cells (*p* < 0.0001) and CH conduit (*p* < 0.005). Interestingly, the CH/NGF-µS/PCL conduit induced monocyte activation when compared to BCS (*p* < 0.005).

Implants with possible application in nerve regeneration or other conduits having contact with blood, tissues, or body fluids should remain noncytotoxic, while they also cannot contain contaminants that may induce pathological inflammation. Bacterial endotoxins (lipopolysaccharides) are microbial components inducing strong inflammation which, in the first stages, target innate immunity cells such as monocytes. Monocytes using Toll-like receptor 4 (TLR-4) recognize and rapidly react to bacterial LPS, and consequently strongly induce nuclear transcription factor (NF-κB) [38]. LPS-induced inflammation has pathological consequences, e.g., in intestinal and endothelial dysbiosis [39] or during sepsis [40]. However, as shown recently, mild activation of these cells is crucial for proper tissue regeneration. The reason for this diverse role in repair is unclear, but inflammation, specifically monocyte activation, likely plays a key role. Monocytes and monocyte-derived macrophages promote the repair of injured sites, including nerve tissue, possibly by regulating transitions through phases of the healing response [41].

In the presented work, it was shown that CH/NGF-µS/PCL, similarly to CH conduit, and the BSC reference material did not contain endotoxin in concentration exceeding the critical level for biomaterials having contact with body fluids (ISO 10993-5:2009). On the other hand, the CH/NGF-µS/PCL conduit exclusively induced low, albeit statistically significant, activation of monocytes, when compared to the reference biomaterial. The monocyte stimulation, in the milieu of additional external signals (such as cytokines, chemokines, and alarmins) transform into macrophages which contribute to all phases of repair: by promoting inflammation, removing injured cells, depositing ECM, stimulating cell proliferation, and releasing anti-inflammatory cytokines that stop the inflammation [41,42]. NGF-mediated monocyte activation was shown to be implicated in spinal cord tissue regeneration [41]. When strongly activated, however, monocytes and monocyte-derived macrophages may interrupt different phases of repair and lead to chronic inflammation and dysfunctional wound healing [43]. Pioneering and recent findings have shown that human monocyte-derived macrophages respond to NGF, and this effect is corelated with morphological and secretory activity, favoring the healing process [44,45].

## 3. Materials and Methods

### 3.1. Materials

Chitosan (CH, type 85/500) was purchased from Heppe Medical Chitosan GmbH (Halle, Germany). Its degree of deacetylation (DD), viscosity (µ, 1% in 1% acetic acid, 20 °C), and viscosity-average molecular weight (Mv) were respectively 82.6–87.5%, 351–750 mPa·s, and 472 kDa. Polycaprolactone (PCL, mean Mn 80,000 mol/wg), dichloromethane (DCM), lactic acid (LA), poly(vinyl alcohol) (PVA), and hydroxyapatite (HAp, nanopowder, <200 nm particle size) were purchased from Merck KGaA (Darmstadt, Germany). Sodium hyaluronate of average molecular weight 2000–2200 kDa was purchased from Contipro (Dolní Dobrouč, Czech Republic). Poly(lactide-*co*-glycolide) (PLGA Resomer^®^ RG 503 H, acid-terminated, lactide/glycolide 50:50) was obtained from Merck KGaA (Darmstadt, Germany). Nerve growth factor (NGF, recombinant mouse protein with an initial Met at the N-terminus) was purchased from Life Technologies (Waltham, MA, USA).

### 3.2. Printing of Polycaprolactone Filament

A four-axis CNC milling machine (Computerized Numerical Control, Shanding U-May Cnc Technology Co., Ltd., Jinan, China) was adapted for working in the vertical plane [8]. The device was described earlier in a patent application to the Patent Office of the Republic of Poland: P. 428594 (2019). The apparatus was equipped with a custom-made 3D print head. The head was built from the following elements: two aluminum blocks, two collaterally connected ceramic heaters of a diameter 6 mm (set at 6 W each), a 0.4 mm diameter nozzle, a thermal barrier with an internal polytetrafluoroethylene layer (connecting aluminum blocks and serving as a casing for the melted filament), and a thermocouple coupled with a PID (proportional–integral–derivative) transmitting G8 controller for keeping a constant temperature of 60 °C. The print head was also equipped with a piston. The piston was made of a 2 mm diameter stainless-steel rod set in motion by the Z-axis motor of the milling machine.

The polycaprolactone filament was produced using a Felfil Evo extruder (Felfil Evo Filament Extruder, Turin, Italy). The extruder operating parameters were set to produce a PCL filament with a diameter of approximately 1.4 mm.

The thermoplastic printing material in the form of the filament was placed in the feeder of a 3D print head. A cylindrical rod made of material consistent with PN-EN ISO 14343 (PN-EN 12072): W 19 9 LSi AWS A5.9: ER 308LSi was used as a build plate. The diameter of the rod can be adjusted to the desired implant dimensions. The presented studies were conducted for a rod with a diameter of 2 mm. Then, the cylindrical rod was set in motion by the A-axis motor of the milling machine. A G-code used to print polymer skeletons was prepared manually during optimization studies and implemented in Mach3 CNC Controller software (version R3.041, ArtSoft Inc., Livermore Falls, ME, USA).

The CNC milling machine was also equipped with a cylindrical stainless-steel reactor for conducing electrophoretic deposition. The inner diameter of the reactor can be adjusted to the desired implant dimensions. The presented studies were conducted using the reactor with an inner diameter of 22 mm.

### 3.3. Preparation and Characterization of Microspheres

Microspheres were prepared using the standard water/oil/water (W/O/W) double emulsion technique with solvent evaporation [25]. Three concentrations of PLGA solution in dichloromethane were prepared: 20%, 25%, and 40%. Then, 0.08 mL of an aqueous protein solution dissolved in PBS containing 0.1% (*w*/*v*) BSA alone or in combination with 0.025 mg of NGF was added to PLGA solution. The mixture was homogenized for 60 s using an ultrasonic homogenizer (Sonoplus HD 2070 homogenizer; Bandelin Electronics GmbH & Co. KG,) operating at 10% power and a cycle count of six. Then, the homogenized mixture was poured to 25 mL of 1 wt.% PVA aqueous solution and homogenized for 1 min using a homogenizer set at power = 50% and number of cycles = 6. Finally, the volume was increased to 25 mL by adding 0.1 wt.% PVA aqueous solution and homogenized for another 1 min using a homogenizer set at power = 50% and number of cycles = 6 for 1 min. Then, the entire mixture was stirred for 1 h on a magnetic stirrer at room temperature. After complete solvent evaporation, the microspheres were centrifuged (2500 rpm for 3 min at room temperature) and washed twice by centrifugation (2500 rpm 3 min at room temperature) in 50 mL of sterile deionized water.

### 3.4. Polycaprolactone Filament Surface Modification

PCL helices obtained for G-code: F8500 Z-240 A17600 Y-132 Z-8 were placed in a solution of 0.2%, 0.4%, or 0.6% dopamine hydrochloride in Tris buffer (0.183 g Tris in 150 mL deionized water with pH adjusted to 8.5 using 0.5 mM hydrochloric acid) [46]. The solutions were subjected to gentle stirring without light for 24 h. Afterward, the PCL helices were removed from the buffer solution and rinsed by immersion in deionized water three times. Then, the PCL helices were placed in a solution containing 2.34% of microspheres and subjected to gentle stirring for 2 h. For the PCL helix modified in 0.4% dopamine hydrochloride, three concentrations of microspheres were applied: 1.19%, 1.5%, and 2.34%.

### 3.5. Conduit Manufacturing

The build plate with PCL helix was placed in a handle of the CNC milling machine described above and aligned straight along the *Y*-axis. Then, the reactor for conducting electrophoretic deposition containing the solution was installed. The chitosan solution concentration was chosen according to previous studies [47]. Briefly, the solution was prepared by dissolving 10 mg of hyaluronic acid in 100 mL of 2.5% (*w/v*) lactic acid. Then, 1 g of chitosan and 0.09 g of hydroxyapatite were added to the solution. The obtained solution was stirred (under slow rotations) until complete dissolution for 24 h. After dissolution, 16 mL of the solution was poured into the cylindrical reactor with an internal diameter of 18 mm. Then, the electrophoretic deposition process was conducted for 15 min at room temperature at 12 V. After the set time, the obtained chitosan/polycaprolactone conduit of average length 40.0 ± 0.2 mm was removed automatically from the reactor and taken manually off from the electrode.

### 3.6. Structural Characterization

#### 3.6.1. Scanning Electron Microscopy

Dry implants were cut into specimens of length 5 ± 0.2 mm for further examination. Scanning electron microscopy photographs of gold-coated deposits were taken with a Hitachi TM-1000 microscope (Hitachi Ltd., Tokyo, Japan).

#### 3.6.2. Fourier Transform Infrared Spectroscopy

The fabricated conduits were dried at 37 °C for 5 days. Samples were examined using a Nicolet™ iS50 FTIR spectrophotometer (Thermo Fisher Scientific, Waltham, MA, USA) with an ATR monolithic diamond crystal mounted. Spectra were recorded in the range of 0–4500 cm^−1^.

#### 3.6.3. Mechanical Testing

Mechanical properties of conduits were investigated by an Instron 3345 apparatus (Instron, USA). The initial length of specimens was 40 ± 1 mm. The ends of samples were inserted on two 5 mm cylindrical internal supports in order to prevent their deformation. Then, the conduit ends were fixed in specially designed clamps. Initially, the distance between the clamps was set to 20 mm. Implants were tested at a strain rate of 3 mm/min at room temperature. Each sample was stretched to the complete tensile failure. A mean value of at least three different measurements was determined, and a standard deviation was calculated.

#### 3.6.4. Water Content

In order to determine the water content, the conduits were divided into two groups: hydrated and dry. The mass of hydrated specimens was measured just after their preparation. To determine the mass of dry specimens, structures were placed at 70 ± 0.1 °C for 24 h. The mass of hydrated and dry conduits was determined after subtracting the mass of the PCL helix. The mean value of three different measurements for both measurements was determined, and the standard deviation was calculated. The water content was determined using the following equation:

(3) $$X=\frac{m_{h}-m_{d}}{m_{h}}\cdot 100\%,$$

where *X* is the water content (%), $m_{h}$ is the mass of the chitosan hydrogel deposit (g), and $m_{d}$ is the mass of the dry chitosan deposit (g).

### 3.7. Protein Controlled Release Studies

In order to assess NGF release kinetics from NGF-µS/PCL helix, 1 cm helices were placed in a solution of phosphate-buffered saline (pH 7.4), bovine serum albumin, and an antibiotic (streptomycin and penicillin). The solutions were subjected to gentle shaking at 37 °C. Released neurotrophic factor was collected by centrifugation of the microparticles at 1000 rpm at room temperature and resuspending in PBS at the following timepoints for quantification via ELISA: 1, 3, 7, and 14 days. The ELISA was carried out as per manufacturer’s instructions (Wuhan Fine Biotech Co., Ltd., Wuhan, China) to determine the cumulative release kinetics of NGF from PCL helices.

### 3.8. Biodegradation Studies

The in vitro degradation was performed in 20 mL of phosphate-buffered solution (PBS, pH 7.4, Merck KGaA, Germany) and in 20 mL of phosphate-buffered solution (PBS, pH 7.4) containing 1.5 mg/mL of lysozyme (human lysozyme, Merck KGaA, Germany). The lysozyme dose was selected on the basis of its physiological concentration in human serum [48]. Briefly, tubular conduits (prepared according to the procedure described in previous section) were incubated in the solution at 37 °C for the period of 1, 7, 14, and 28 days. The lysozyme solution was refilled three times per week to maintain enzyme activity. After 1, 7, 14, and 28 days samples were removed from the medium, dried at 70 °C for 48 h, and weighed. A mean value of at least four different measurements (i.e., four independent samples) was determined, and a standard deviation was calculated.

### 3.9. Biological Properties of the Conduit

#### 3.9.1. Cell Culture and Preparation for the Biological Assays

The hippocampal mouse cells (mHippoE-18, CELLutions Biosystems, Ontario, Canada) were routinely maintained in DMEM high-glucose culture medium (Sigma-Aldrich, Missouri, MO, USA) without sodium pyruvate. The THP1-Blue™ NF-κB cells derived from the human monocytic THP-1 cell line (Invivogen, Toulouse, France) were maintained in RPMI 1640 medium (Cytogen, Zgierz, Poland). Media for both cell lines were supplemented with 10% fetal bovine serum (Biowest, Nuaille, France), 100 U/mL penicillin, and 100 μg/mL streptomycin (Penicillin-Streptomycin Solution ATCC^®^). In addition, the medium for THP1-Blue™ NF-κB was supplemented with 100 μg/mL normocin (InvivoGen, San Diego, CA, USA), and 10 μg/mL blastocidin (InvivoGen, USA). The mHippoE-18 cells were trypsinized (Trypsin-EDTA Solution, ATCC^®^) twice a week, seeded at a density of 5 × 10^5^ cells per T25 cell culture flask, and incubated at 37 °C and 5% CO_2_ to obtain a confluent monolayer. The THP1-Blue™ NF-κB cells were also incubated at 37 °C in a humidified incubator with 5% CO_2_ and passaged every 3 days to maintain density < 2 × 10^6^ cells/mL.

#### 3.9.2. Direct Contact Cytotoxicity Assay

The CH and CH/NGF-µS/PCL conduits were tested for cytotoxic properties upon direct contact with mHippoE-18 embryonic hippocampal cells, as described previously [26]. Briefly, the hippocampal cells were seeded in the 96-well plate at a density of 2 × 10^4^ cells/well and incubated for 24 h. Tested implants were cut into fragments constituting 1/10 of the well surface and placed carefully on the cell monolayer. After 24 h of incubation, the MTT reduction assay was used to determine the viability of the cells. The viability was calculated in comparison to cells grown in the presence of the commercially available biomaterial (BCS, Safety-Lok™ blood collection set, BD, Franklin Lakes, NJ, USA), which served as a positive control of viability (100%). Hydrogen peroxide 3% (Sigma-Aldrich, Missouri, MO, USA) was used as a negative control of viability.

#### 3.9.3. Cell Proliferation

To determine the influence of CH/NGF-µS/PCL conduit on mHippoE-18 cell proliferation, we performed an assay where 0.2 mL of hippocampal cell suspension (5 × 10^4^ cells/mL) was seeded in a 96-well tissue culture plate (Corning, Wilmington, NC, USA); then, after 24 h of incubation, the conduit fragments constituting 1/10 of the well surface were placed carefully on the cell monolayer. After another 24 h of incubation, DNA content was measured using a CyQUANT^®^ Cell Proliferation Assay Kit (Thermo Fisher Scientific, Waltham, Massachusetts). The wells with samples were washed with PBS and then frozen at −80 °C. Next the plate was thawed at room temperature, and samples were lysed in buffer containing the CyQuant-GR dye, which bound to cellular nucleic acids. Fluorescence was measured using SpectraMax^®^ i3x Multi-Mode Microplate Reader (Molecular Devices, San Jose, CA, USA) at E_m_ = 520 nm and E_x_ = 480 nm. The commercially available biomaterial Safety-Lok™ blood collection (BCS) served as a negative control.

#### 3.9.4. Cell Morphology

To evaluate the influence of CH/NGF-µS/PCL conduit on mHippoE-18 cells, we performed an assay where 0.2 mL of hippocampal cell suspension (5 × 10^4^ cells/mL) was added on the biomaterial placed in 96-well tissue culture plates (Corning, Wilmington, NC, USA). After 48 h incubation, cells propagating near biomaterial were analyzed, and their axonal length was measured with the use of inverted microscope (IX50 microscope with UC90 camera, Olympus, Tokyo, Japan). To evaluate the average axonal length, microscopic analysis of at least 100 cells was performed using cellSens software (cellSens Standard 2.3, Olympus, Tokyo, Japan).

#### 3.9.5. Quantification of NF-κB Induction

To address the question whether the obtained conduits activate monocytes, we used the THP1-Blue™ NF-κB reporter cell line, which enables to quantify the induction of NF-κB transcription factor in monocytes. The test was performed according to the procedure described previously [49]. Briefly, the THP1-Blue™ NF-κB cells were seeded in 24-well plates at a density of 1 × 10^6^ cells/mL; then, the conduits, cut into fragments constituting 1/10 of the well surface, were introduced to each well and incubated for 24 h. Next, 20 μL of supernatants from monocyte cultures were added to 200 μL of Quanti-Blue™ reagent, and the quantification of alkaline phosphatase, as a marker of cell activation (inflammatory response), was measured at 650 nm. The following controls were included: untreated monocyte cultures served as a negative control (cRMPI) and cultures treated with *Escherichia coli* (*E. coli*) lipopolysaccharide (LPS) (Sigma–Aldrich, USA) served as a positive control. Furthermore, the commercially available biomaterial Safety-Lok™ blood collection (BCS) served as a reference polymer. Experiments were independently repeated three times, and at least four repeats were included each time.

### 3.10. Statistical Analysis and Graphs

The results were subjected to statistical analysis with the use of GraphPad Prism version 9.0.0 on macOS (GraphPad Software, San Diego, CA, USA). Following the confirmation of data normality, the statistical significance of differences was evaluated in ANOVA test, and, for significant comparisons, further analysis was performed using the Tukey’s multiple comparisons test. The differences were considered significant for a *p*-value < 0.05.

## 4. Conclusions

A new approach for the incorporation of biologically active agent-loaded microspheres into the structure of chitosan/polycaprolactone conduits was described. Internal PCL helical thread was used to mechanically stabilize the conduit and provide the support for microspheres releasing the biologically active substances. The chitosan hydrogel tube produced by electrodeposition is perfectly suited for application as a nerve guidance conduit due to high water content and softness, mimicking the surrounding soft tissue. Upon incorporation of chitosan deposit with PCL helix, the structure provides space and environment for regrowing nerves and accumulation of signaling molecules, preventing infiltration with inflammatory cells and formation of fibrous tissue. The obtained results indicate that the developed strategy enables a spatiotemporal controlled release of nerve growth factors as the helix dimensions and geometry can be customized.

The structural properties of implants during incubation in phosphate-buffered solution (pH 7.4) did not change significantly up to 28 days at 37 °C. Furthermore, implants manufactured by our technology are cytocompatible with neuronal cells, stimulate axonal growth of hippocampal cells due to controlled release of NGF, and activate monocytes via NF-κB transcription factor. These complex in vitro results give us a strong argument for further examination of the implants in an appropriate in vivo animal model. As peripheral nerve injuries represent a significant problem to be solved, the developed biologically active conduits contribute to the state of the art, providing solutions for conduit manufacturing and the release of biologically active molecules required for efficient regrowth of the discontinued nerves.

## 5. Patents

A patent application was sent to the Patent Office of the Republic of Poland: P. 438938 (2021): A method of producing hybrid implants with a cylindrical shape for the controlled release of active substances.

## Data Availability

The data presented in this study are available on request from the corresponding author.
